# Differential expression of host invasion-associated genes by *Sarcocystis calchasi* in intermediate versus definitive hosts

**DOI:** 10.1371/journal.pone.0322226

**Published:** 2025-06-03

**Authors:** Kristina Maier-Sam, Oliver Rupp, Anne Voss, Saskia Nemitz, Tassilo E. Wollenweber, Tara Procida-Kowalski, Jochen Wilhelm, Achim D. Gruber, Michael Lierz

**Affiliations:** 1 Clinic for Birds, Reptiles, Amphibians and Fish, Justus Liebig University, Giessen, Germany; 2 Department of Bioinformatics and Systems Biology, Justus Liebig University, Giessen, Germany; 3 Institute of Veterinary Pathology, Freie Universität Berlin, Berlin, Germany; 4 Biological and Medical Research Center (BMFZ), Genomics & Transcriptomics Laboratory, Medical Faculty, Heinrich Heine University, Düsseldorf, Germany; 5 Institute for Lung Health (ILH), Genomics and Bioinformatics, Justus Liebig University, Giessen, Germany; Guru Angad Dev Veterinary and Animal Sciences University (GADVASU), INDIA

## Abstract

*Sarcocystis calchasi* is a pathogenic apicomplexan parasite affecting avian species of several orders. To complete its heteroxenous life cycle, *S. calchasi* infects a wide range of avian intermediate hosts and accipitriform raptors serve as definitive hosts. The mechanism of invasion into host cells is largely understood in other apicomplexan parasites, particularly *Toxoplasma gondii*, which also belongs to the family of Sarcocystidae. However, *Sarcocystis* species exhibit several distinguishing features in their life cycles and in their secretory organelles. The composition of secretory pathogenesis determinants, including surface antigens and secretory organelle proteins, has been shown to differ between closely related species, as evidenced by *Sarcocystis neurona*. In this study, whole-genome sequencing was performed on *S. calchasi*, and transcriptomes were determined by RNA-seq of *S. calchasi* sporozoites and bradyzoites derived from intermediate and definitive hosts as well as from merozoites propagated in primary embryonal pigeon liver cells. The *S. calchasi* genome contains homologs of genes encoding proteins associated with the well-conserved host invasion machinery like AMA1 and rhoptry neck proteins, albeit with a markedly reduced number of genes encoding surface antigens, rhoptry and dense granule proteins in comparison to *T. gondii*. Our transcriptome analysis revealed different gene expression profiles between *S. calchasi* sporozoites, merozoites and bradyzoites. Factors associated with host cell attachment (surface antigens and micronemal proteins) were expressed predominantly either in sporozoites and merozoites or in bradyzoites. As sporozoites and merozoites invade various intermediate hosts and cell types whereas bradyzoites enter definitive host intestinal epithelium, their differential expression patterns indicate that *S. calchasi* utilizes different sets of secretory pathogenesis determinants for host cell attachment and invasion, depending on the type of host and cell.

## Introduction

*Sarcocystis calchasi* is an apicomplexan parasite and the etiological agent of a neurologic disease in pigeons and various other avian species. *Sarcocystis calchasi* was discovered in 2009 in racing pigeons (*Columba livia* forma *domestica*) suffering from pigeon protozoal encephalitis (PPE) [1]. The northern goshawk (*Accipiter gentilis*) had initially been identified as definitive host of *S. calchasi* [2], followed by other members of the order Accipitriformes (*Accipiter nisus, A. cooperi, Buteo jamaicensis*) that have been established as definitive hosts so far [3,4]. The spectrum of intermediate hosts, on the other hand, encompasses species of several avian orders. These include domestic and feral pigeons and doves (Columbiformes) [5–8], various psittacine species (Psittaciformes) [9,10], Brandt`s cormorants (Suliformes) [11], woodpeckers (Piciformes) [12], and a vulturine guineafowl (Galliformes) [13], most developing PPE-like disease. Reports of sporadic outbreaks of *S. calchasi*-associated diseases originated from Europe, North America, and Asia and were often associated with large numbers of affected animals or increased wildlife casualties [1,11,14,15].

Parasites of the genus *Sarcocystis* are characterized by three distinct developmental stages that invade host cells. Following ingestion of infectious sporocysts from the definitive host, sporozoites are released from the sporocysts in the intestines of intermediate hosts, enter small intestinal blood vessels and migrate to target cells [16]. In the case of *S. calchasi*, schizogony occurs mainly in liver and spleen [17,18]. After asexual reproduction, merozoites are set free from disrupted host cells and invade either neighboring cells for repeated schizogony or migrate to muscular and central nervous tissue for cyst formation in host cells. The third invasive stage are bradyzoites that are set free from the muscular sarcocysts after their ingestion by definitive hosts where they invade intestinal epithelial cells [16]. In closely related apicomplexans like *T. gondii*, *Neospora caninum*, and *S. neurona*, the invasion process is mediated by several sets of proteins which can be summarized as secretory pathogenesis determinants (SPDs) [19]. Host cell recognition and attachment is mediated by highly regulated glycosylphosphatidylinositol-anchored proteins, termed surface antigens (SAGs) or SAG-related sequences (SRS) [20,21]. Their number varies greatly between different species and even between different strains. In *T. gondii*, 109 functional SRS genes have been identified in the ME49 strain and 91 in the GT1 strain [20]. With 227, *N. caninum* possesses more than twice the number of functional SRS genes, even though the number of simultaneously expressed SRS genes was lower than that in *T. gondii* [22]. The *Sarcocystis neurona* SN1 strain genome encodes for 23 SRS genes [23] with at least six additional SRS-domain containing genes in the S3 strain [24].

Host cell invasion is orchestrated by several proteins from micronemes and rhoptries. Micronemes are lancet-shaped secretory organelles unique to apicomplexans located in the apical region of all invasive stages. Micronemal proteins (MIC) are discharged immediately after host cell attachment. They are transferred to the cell surface by fusion with the cell membrane [25]. For host cell invasion, apicomplexans establish a moving junction, a tight connection between the parasite and the host cell. During the process of invasion, the ring-shaped moving junction moves towards the posterior pole of the parasite until its internalization into the host cell is completed. The moving junction is formed by the micronemal protein AMA1 (apical membrane protein 1) and four rhoptry neck proteins, RON2, RON4, RON5 and RON8. AMA1 and RON proteins form a macromolecular complex with AMA1 being linked to RON2 [26]. These proteins are highly conserved among apicomplexans with intracytoplasmic development (*Toxoplasma*, *Neospora*, *Plasmodium*, *Eimeria*, *Theileria*, and *Babesia*) and have also been found in *S. neurona* [23,27]. In *T. gondii*, further paralogs of AMA1 and RON2 have been associated with different developmental stages. While AMA1 and RON2 seem to play a predominant role in tachyzoite invasion, AMA3, or sporoAMA1, and RON2_L2_, or sporoRON2, link similarly at the sporozoite apical tip during invasion [28]. AMA4 and RON2_L1_ are both highly expressed in bradyzoites as well as in sporozoites but not in tachyzoites. Their linking seems to play a crucial role for bradyzoite host cell invasion [29].

Essential for host cell invasion in *T. gondii* is also a complex formed by cysteine repeat modular proteins, including CRMPA and CRMPB, as well as MIC15 and thrombospondin type-1 domain-containing protein, TSP1. This complex seems to play an important role in host cell invasion in *T. gondii* tachyzoites as disruption of the complex leads to a block of rhoptry secretion and to markedly reduced host cell invasion [30]. CRMPs are present in all apicomplexans with low sequence conservation, and CRMP motifs can be found even in alveolata. Apicomplexans usually possess two CRMPs while in free-living alveolata like *Chromera velia* and *Paramecium tetraaurelia* a highly expanded set of CRMPs was identified [30]. In *T. gondii*, CRMPA and CRMPB are located subcellularly throughout the secretory pathway with a transient exocytic appearance immediately prior to host cell invasion [31].

While micronemal proteins and proteins from the rhoptry neck are the main actors of host cell invasion, proteins from the rhoptry bulb and dense granular proteins are mostly associated with the establishment of the intracellular niche and the interaction with the host immune system. Rhoptry bulb proteins (ROP), a family of kinases and pseudokinases, are associated predominantly with host cell modification. Rhoptry proteins are secreted after invasion in a second wave after microneme discharge. In *T. gondii*, rhoptry proteins are injected directly into the host cell cytosol, mediate host cell immune response [25] and are involved in the establishment of the parasitophorous vacuole (PV) [32]. Dense granules are secretory vesicles containing a large set of proteins in *T. gondii*, *N. caninum* and other apicomplexans. These proteins interact with host cell innate immune response, e.g., activation of M1 macrophages in *T. gondii* type II strain by GRA15 [33] and support the maintenance of the PV [34].

Another structure in apicomplexans associated with parasite motility, host invasion and replication is the inner membrane complex (IMC). The IMC consists of flattened alveolar sacs supported by a network of alveolin-domain containing proteins. The IMC is sandwiched between the plasma membrane and subpellicular microtubules. Acting as anchor for the actin-myosin motor system, the IMC is involved in the parasite’s gliding motility and active host cell invasion [35]. IMC proteins in *T. gondii* are a heterogeneous group of >40 proteins [36]. Apart from the alveolins that provide stability as part of the cytoskeleton, other IMC proteins are located at different sub compartments of the IMC [37] and are expressed in different developmental stages and at different phases of cell division [38].

While in *Toxoplasma* the expression of the SPDs is often stage-specific, little is known for the genus *Sarcocystis*. *Sarcocystis neurona,* which is pathogenic for numerous mammalian species, expresses several SPDs with marked differences between intracellular schizonts and extracellular merozoites [24]. Moreover, SAGs have been shown to be differentially expressed in bradyzoites and sporozoites [39]. In *S. calchasi,* it is unknown whether the same homologs to *T. gondii* SPDs exist as in *S. neurona* and if they are expressed in a stage-specific fashion. Of note, *Sarcocystis* spp. developmental stages possess differences in their setup of secretory organelles and their intracellular niche compared to the corresponding stages in the *T. gondii* life cycle. For instance, merozoites lack rhoptries and schizogony does not occur within a PV but in the host cell cytoplasm [16]. Therefore, differences in SPD gene expression can be expected. Identification of stage-specific gene expression patterns may thus help to identify virulence factors in the genus *Sarcocystis*. In the present study, we identified and characterized SPD-related genes in the *S. calchasi* genome and their expression in different stages of the *S. calchasi* life cycle.

## Materials and methods

### Ethics statement

All experiments that involved live animals were conducted with the approval of local animal welfare authorities. The experiments were reviewed and approved by the Regierungspräsidium Giessen (approval numbers: GI 18/5 A7/2018 and GI 18/5 G32/2018). All animals were euthanized by exsanguination under general anesthesia with isoflurane.

### Parasites

*Sarcocystis calchasi* sporocysts were obtained from an experimentally infected northern goshawk. The goshawk originated from the wild and was presented to the Clinic for Birds, Reptiles, Amphibians and Fish at the Justus Liebig University Giessen with clinical conditions impeding the release back to the wild. The goshawk’s feces from three consecutive days were tested for the absence of *Sarcocystis* spp. sporocysts by flotation in saturated saline three times over a period of 40 days. The goshawk was infected by feeding the carcass of a pigeon experimentally infected with *S. calchasi* isolate Giessen16. This was done as part of the regular host passage for maintenance of the isolate. Fourteen days after infection (dpi) the goshawk was euthanized, its intestines scraped and sporocysts isolated from the gut mucosa and intestinal content via trypsin digestion and filtering. The sporocyst suspension was finally treated with sodium hypochlorite, counted in a hemocytometer and stored in Hank’s Balanced Salt Solution (HBSS) supplemented with penicillin/streptomycin and amphotericin B at 4°C.

To obtain sporozoites, sporocysts were excysted via bile incubation. Briefly, sporocysts were washed once with PBS, treated shortly for 10 min with sodium hypochlorite, washed five times and incubated for 4.5 h in Dulbecco’s minimal essential medium high glucose with 3% fetal bovine serum, glutamine and penicillin/streptomycin (DMEM culture medium) containing 15% chicken bile. Released sporozoites were washed three times with DMEM culture medium and counted in a hemocytometer.

*Sarcocystis calchasi* bradyzoites were isolated from the pectoral muscle of a pigeon experimentally infected with the same sporocysts as used for sporozoite extraction as described by Verma 2017 [40]. Pectoral muscle was homogenized with a commercial blender, digested with HCl-pepsin solution and filtered through sterile gauze. After removal of host cell debris, bradyzoites were washed with PBS and resuspended in HBSS supplemented with penicillin/streptomycin and amphotericin B. Bradyzoites were counted in a hemocytometer and stored in 4°C or liquid nitrogen.

### Cell culture

To produce merozoites, cell cultures were infected with sporozoites. After repeated testing of permanent cell lines (Vero cells, obtained from the German Collection of Microorganisms and Cell Cultures GmbH, ACC 33, and CEC-32 [41]) with very inconsistent success rates, primary pigeon liver cells were used for culturing *S. calchasi* merozoites. Primary cells were produced by incubation of fertilized pigeon eggs in an incubator at 37°C, approx. 60% humidity and regular turning. On day 12, eggs were removed from the incubator. The eggshell was opened aseptically and the embryo removed. After decapitation, the liver was separated from the embryo, washed twice in PBS and digested in trypsin solution for 30 min or until the tissue had dissolved. The suspension was filtered through sterile gauze, centrifuged for 2 min at 2,000 x *g* and resuspended in 6 ml DMEM culture medium. The cells were incubated at 37°C with 5% CO_2_ for 1–3 days until a monolayer had developed.

Sporocysts were excysted as described above with additional removal of remaining sporocysts in a PD desalting column. The final pellet of sporozoites was taken up in 200 µL DMEM culture medium and dispensed in the supernatant of the primary pigeon liver cells. Appearance of merozoites in the supernatant was monitored every 2–3 days. If a considerable number of merozoites was observed, the supernatant was removed, passed through a PD desalting column, and merozoites were counted in a hemocytometer and either processed directly or stored in liquid nitrogen. The cells were covered with fresh DMEM culture medium and re-incubated until no further parasite development was observed.

### DNA and RNA extraction

High molecular weight DNA was extracted from 2 x 10^8^ bradyzoites using a phenol/chloroform protocol [42]. RNA was extracted from freshly excysted sporozoites, merozoites from cell culture supernatant, and freshly isolated bradyzoites with the RNeasy Mini Kit (Qiagen, Hilden, Germany) with additional on-column DNase treatment (RNase-Free DNase Set, Qiagen). In total, three replicates of sporozoites (6.8 x 10^5^–3.23 x 10^6^) and both four replicates of merozoites (1.6 x 10^5^–2.11 x 10^6^) and bradyzoites (1.6 x 10^9^) were processed. Extracted RNA amount and quality were assessed photometrically with the NanoDrop 2000c (Thermo Fisher Scientific, Wilmington, DE, U.S.A.), followed by immediate freezing at -80°C.

### Whole genome sequencing

The library was prepared using the ultra-low input whole genome sequencing workflow according to protocol version 02 (“Preparing HiFi SMRTbell Libraries from Ultra-low DNA Input (PacBio, Version 02; August 2020)), with adaption to the SMRTbell® Prep Kit 3.0 (PacBio, Pacific Biosciences, CA, U.S.A.). In summary, 20 ng of DNA was sheared using a g-TUBE (Covaris, MA, U.S.A.), with a centrifugation program with incremental speed settings aiming at a mean fragment size of 12–15 kb. The fragment size distribution was quality controlled by running a dilution of the sheared sample on a Femto Pulse (Agilent, Agilent Technologies, CA, U.S.A.) with FP-1002 run protocol (165 kb). After assessing the size distribution, the sheared sample was used as input for the library preparation according to the manufacturers’ instructions. In short, the libraries were purified using 0.8X ProNex beads (Promega, Madison, U.S.A.) and eluted in 49 µl elution buffer after incubation at 37°C for 20 min. A whole genome amplification was performed with the gDNA Sample Amplification Kit (Pacific Biosciences, CA, U.S.A.) according to the above-mentioned protocol with the following modifications: The elongation time for both PCR programs (Reaction mix A and Reaction mix B) were increased to 10 min and used for 15 cycles. The concentration of the amplified library was determined with the Qubit dsDNA HS Assay (Thermo Fisher Scientific, MA, U.S.A.) and size distribution was analyzed on a Fragment Analyzer (Agilent) with DNF-464 run protocol using a 1 ng/µl dilution in TE. The amplified libraries of the two reaction mixes were pooled and a second library preparation was performed with the SMRTbell Prep Kit 3.0 (PacBio) following the protocol version 02 (Procedure-checklist-Preparing whole genome and metagenome libraries using SMRTbell prep kit 3.0, PacBio; Version 01, April 2022) with the modification of increasing the ligation time of the SMRTbell adapters to 60 min. A nuclease treatment was performed to remove non-circular library templates and the library was size-selected on a BluePippin (Sage Science, MA, U.S.A.) with the following settings: 0.75% DF Marker S1 High-Pass 6–10 kb vs3 using a cutoff at 6 kb. The size-selected library was purified with SMRT bell cleanup beads (PacBio) and eluted in 11 µl elution buffer. The concentration and size distribution of the library were assessed with the Qubit dsDNA HS kit (Thermo Fisher Scientific) and a Fragment Analyzer (Agilent), respectively. Sequencing primer (v3.2) and polymerase were bound to the library using the Sequel® II Binding Kit 3.2 (PacBio) and the bound library was sequenced on a Sequel IIe instrument using a Sequel II SMRT Cell (8M) with a final on plate loading concentration of 85 pM, 2 hrs pre-extension and 30 hrs movie time. The circular consensus reads were generated with SMRT Link version 11, using a minimum accuracy of 0.99 and 3 passes.

### RNA sequencing

A total of 25 ng of RNA per sample was used to enrich for polyadenylated mRNA followed by cDNA sequencing library preparation utilizing the Illumina^®^ Stranded mRNA Prep Kit (Illumina, San Diego, CA, U.S.A.) according to the manufacturer’s instructions. After library quality control by capillary electrophoresis (4200 TapeStation, Agilent), cDNA libraries were sequenced on the Illumina NovaSeq 6000 platform generating 150 bp paired-end reads.

### Genome assembly and annotation

First, a k-mer spectrum for the PacBio HiFi reads was computed using the Jellyfish 2 [43] modules count and histo with a k-mer size of 25 in canonical mode. According to the single peaked k-mer spectrum, a hifiasm [44] assembly was carried out with the options --primary --n-hap 1 --hom-cov 80. The resulting contigs were screened for assembly duplication using the “comp” module of the k-mer analysis toolkit KAT [45] and BUSCO [46] with the coccidia database (coccidian_odb10).

The genome annotation was done in several steps. First, a repeat library was constructed using RepeatModeler [47], then the assembled contigs were soft-masked with the repeats using RepeatMasker [48]. The RNA-seq reads were merged using FLASH [49] (-M 100 -O) and aligned to the contigs using STAR [50] in two-pass mode. Reference genomes and proteomes of 11 *T. gondii* strains and 2 *S. neurona* strains were downloaded from ToxoDB (Release 67) [51]. Then, three different methods for gene model predictions were computed. First, a de novo prediction was computed using BRAKER3 [52] including the RNA-seq read mappings and the ToxoDB proteomes. Second, the ToxoDB annotations were used to compute a homology-based prediction using GeMoMa [53]. For the third prediction, the RNA-seq mappings were assembled separately using StringTie 2 [54] and then merged with the StringTie-merge option. The three gene models were combined using EVM [55] with different weights (BRAKER3 = 4, GeMoMa = 2, StringTie = 1). Quality of the final model was assessed using BUSCO with the coccidia database.

The functional annotation of the gene model was derived from best blast hits against the ToxoDB proteomes using DIAMOND [56] (blastp --iterate --evalue 10^-20^) and an InterProScan [57] search including GO terms. Gene families were identified by a best bidirectional blastp approach of the predicted gene models against reference genes from *T. gondii*.

The Orthofinder algorithm [58] was applied to identify groups of orthologous genes (orthogroups) between *S. calchasi*, *T. gondii* (ToxoDB) and *S. neurona* (ToxoDB). The orthogroups were used to identify syntenic blocks between the three genomes. DIAMOND blastp results of the orthologous genes were re-computed and the MCScanX tool [59] was applied to compute the syntenic blocks. Orthogroups and species-specific genes of *S. calchasi*, *S. neurona* and *T. gondii* were counted with BioVenn [60].

All PacBio and Illumina sequencing datasets and their assembly dataset were deposited at the European Nucleotide Archive (ENA) with the study identifier PRJEB77870. Within this study, the PacBio dataset can be accessed under the accession numbers ERS20600716 (raw data) and ERS20818611 (assembled genome). RNA-seq data of bradyzoites are available under accession numbers ERS20818612, -13, -14, and -86, data of merozoites under ERS20818687, -88, -89, and -90, and data of sporozoites under ERS20818705, -739, and -890.

### Differential gene expression analysis

The read counts and TPM (transcripts per million) values were estimated using Salmon (v2) [61]. The Salmon index was constructed using decoy sequences according to the Salmon manual. Differentially expressed genes were detected using pair-wise comparisons of the samples using the DESeq2 algorithm [62]. The appropriateness of the assumptions underlying the DESeq2 normalization was evaluated using the degCheckFactors function of the DEGreport R module [63]. Genes with a log2 fold change greater than 1 or smaller than -1 and an FDR adjusted p-value below 0.01 were considered differentially expressed.

### *In situ*-hybridization

Four SRS mRNA sequences (ScSRS4, ScSRS8, ScSRS10, and ScSRS12) were localized on microscopical tissue sections by *in situ*-hybridization. Samples were prepared from liver and pectoral muscle of experimentally infected pigeons. Liver tissue was collected from pigeons in the acute (schizogonic) phase of PPE at dpi 10, pectoral muscle from pigeons in the chronic (cyst-bearing) phase of PPE at dpi 59. *In situ*-hybridization was performed using the RNAscope 2.5 HD Reagent Kit-Red (Advanced Cell Diagnostics, U.S.A.) according to manufacturer´s instructions. In brief, formalin-fixed, paraffin embedded tissue sections of 5 µm were deparaffinized with xylene and rehydrated with graded ethanols. For liver samples, hydrogen peroxide was applied for 10 min at room temperature. As minor adjustment from the protocol, muscle samples were incubated with Levamisole (Vector Laboratories, Burlingame, U.S.A.) instead, followed by washing in distilled water. Slides were boiled in RNAscope Target Retrieval Reagents for 15 min. After applying a hydrophobe barrier, slides were incubated with supplied Protease Plus for 30 min at 40°C in a wet chamber. Each of four custom-designed probes targeting ScSRS8, ScSRS4, ScSRS10, ScSRS12 (Table 1) was hybridized for 2 hrs at 40°C followed by multiple amplification steps. A probe targeting the mRNA of dihydrodipicolinate reductase (DapB) was used to control for unspecific binding. For bright field evaluation a red signal was achieved by using a Fast Red chromogen. Slides were counterstained using Mayer’s hematoxylin.

**Table 1:** Probes for ScSAG4, ScSAG8, ScSAG10, and ScSAG12 mRNA.

Probe	Catalog Number ACD	Gene ID, target region and sequence (5’-3’)
RNAscope® Probe - P-S.calchasi-SRS4-C1	1277221-C1	Gene ID: ptg000004l.823 Target region bp: 2–857 Excerpt: TGTTA…TTCCT
RNAscope® Probe - P-S.calchasi-SRS10-C1	1277231-C1	Gene ID: ptg000005l.213 Target region bp: 3–808 Excerpt: GACGA…TGGCA
RNAscope® Probe - P-S.calchasi-SRS8-C1	1277241-C1	Gene ID: ptg000001l.476 Target region bp: 2–486 Excerpt: TGGCG…TCGCC
RNAscope® Probe - P-S.calchasi-SRS12-C1	1277251-C1	Gene ID: ptg000011l.377 Target region bp: 2–801 Excerpt: TGACA…CCACA

### qPCR

For confirmation of RNA-seq data, the mRNA expression of selected surface antigens (ScSRS4, ScSRS8, ScSRS9, ScSRS10, ScSRS12, ScSRS15), rhoptry bulb (ScROP9, ScROP20, ScROP29, ScROP30) and rhoptry neck (ScRON2, ScRON2_L1_, ScRON9) proteins, and microneme proteins (ScMIC2, ScMIC12, ScMIC13, ScMIC15, ScAMA1a, ScAMA1b, ScAMA4) was quantified by RT-qPCR in RNA isolated from sporozoites, merozoites and bradyzoites. Primers were designed with Primer–BLAST [64] for the selected genes as well as potential reference genes (S1 Table). Two reference genes were selected that showed similar expression levels in all three stages. Templates of cDNA were amplified with HotStarTaq DNA Polymerase (Qiagen). Efficiency of primers was determined by triplicate testing of serial dilutions of *S. calchasi* gDNA. Each gene was tested simultaneously with 2 reference genes in triplicate in 3 replicates of sporozoite and merozoite RNA and 4 replicates of bradyzoite RNA. Differential gene expression was calculated by dCt analysis with adjustment of PCR efficiency [65] by qPCRsoft software (Analytik Jena, Jena, Germany). The genes were clustered by hierarchical clustering using the Eucledian distance metric and average linkage. Differences between dCt values were statistically analyzed with the unpaired student’s t-test.

## Results

### *Sarcocystis calchasi* replicates in primary pigeon embryo liver cells

Propagation of *S. calchasi* in permanent cell lines was successful in Vero cells once but, after passages of the parasite through its natural hosts, further attempts were unsuccessful. Likewise, the avian cell line CEC-32 (quail fibroblasts) could not be infected by *S. calchasi* sporozoites. In contrast, primary pigeon embryo liver cells and their surrounding fibroblasts were readily and reproducibly infected. Successful infectious doses for 25 mL flasks ranged from 1.04 x 10^5^ to 1.7 x 10^6^ sporozoites. Large numbers of free merozoites in the supernatant (6.77 x 10^5^–2.11 x 10^6^) were observed from dpi 5 onwards and could be maintained until dpi 13 the longest. Thereafter, the release of merozoites declined and around dpi 21 the monolayer of primary cells dissolved. Attempts to passage free merozoites onto fresh primary cells were unsuccessful.

### The *S. calchasi* genome resembles the *S. neurona* genome with few genes unique to the genus *Sarcocystis*

**Fig 1:**
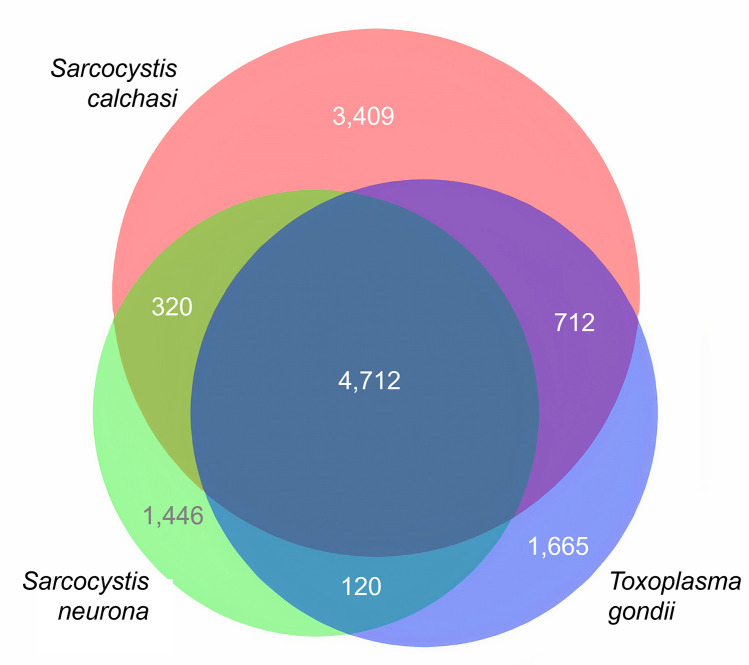
Core genome of *Sarcocystis* spp. and *T. gondii.* Euler diagram of orthogroups shared by *S. calchasi* (red), *S. neurona* (green) and *T. gondii* (blue). Figures refer to the numbers of orthogroups with unique or overlapping presence.

**Fig 2:**
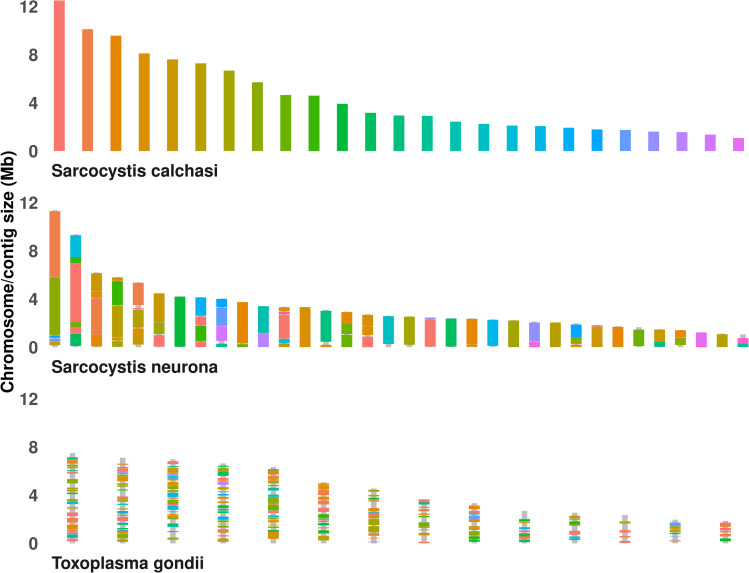
Synteny between *S. calchasi*, *S. neurona* and *T. gondii* genomes. *S. calchasi* (top) and *S. neurona* (middle) scaffolds are in large parts syntenic reflecting their close relationship. In comparison, *T. gondii* (bottom) chromosomes do not share large syntenic parts with *Sarcocystis* spp. scaffolds.

PacBio platform Sequel IIe generated 34.72 Gb sequence data from 3,046,204 reads encoded in 75 contigs. The assembly N50 value was 6 Mbp with a maximum contig length of 12.50 Mbp (mean length: 1.52 Mbp). The k-mer spectrum showed a single peak, which indicates a haploid or a highly homozygous genome. The size of the assembled genome of 113.89 Mbp is similar to the *S. neurona* SN1 genome (127 Mbp) and roughly twice the size of the *T. gondii* genome (ME49: 65.67 Mbp). Like in *S. neurona*, large parts of the *S. calchasi* genome consist of repetitive elements (30.11 Mbp) whereas the *T. gondii* genome includes as few as 2.5 Mbp. Quality of genome assembly was assessed with KAT and BUSCO analysis. Both analyses did not show any significant duplication. Of 502 BUSCO groups in the lineage dataset of 20 coccidia genomes, 495 BUSCOs (98.6%) were complete indicating an almost complete assembly. Gene annotation comprised several methods (see Material and methods) and included RNA-seq data from 11 *S. calchasi* samples (4 x bradyzoites, 4 x merozoites, 3 x sporozoites) with an average size of 80.69 Mio. reads. From these data, 9,716 coding sequences (CDS) were predicted, 118 rRNA sequences and 89 tRNA sequences. For 6,730 CDS homologs were found in the ToxoDB database and 4,609 InterPro hits were generated. In total, 6,508 CDS were functionally annotated which is similar to *S. neurona* with 6,938 genes annotated in the SN3.E1 *S. neurona* genome. Quality of annotation was again assessed by BUSCO analysis in protein mode. Of 502 BUSCO groups, 497 (99.0%) were complete. Orthofinder identified a large core genome in the family Sarcocystidae with 4,712 shared orthogroups in *Toxoplasma* strain ME49, *S. neurona* strain SN3 and *S. calchasi* isolate Giessen16. The number of orthogroups shared by *T. gondii* and *S. calchasi* (712) is more than twice the number shared by *S. neurona* and *S. calchasi* (320) and the number of genes unique to *S. calchasi* (3,409) is more than twice the number of unique genes in *S. neurona* (1,446) and *T. gondii* (1,665) (Fig 1). To evaluate the accuracy of gene prediction in the different sets of unique and shared genes, the percentage of *S. calchasi* genes with TPM <1 was assessed, averaged over all samples (Table 2). This revealed a large number of genes that were either low expressed or erroneously predicted in genes unique to *S. calchasi* (40.00%) or shared by *S. calchasi* and *T. gondii* (34.02%). Regarding the ordering of homologous genes, *S. calchasi* and *S. neurona* genomes share large syntenic blocks suggesting a high level of common chromosome arrangement (Fig 2). In contrast, *S. calchasi* and *T. gondii* genomes are much less syntenic as it has been observed for *S. neurona* and *T. gondii* [23].

**Table 2:** Percentage of *S. calchasi* genes with TPM <1, averaged over all samples.

Presence of genes			Percentage of *S. calchasi* genes with TPM <1
*S. calchasi*	*S. neurona*	*T. gondii*	
+	–	–	40.00%
+	–	+	34.02%
+	+	–	11.75%
+	+	+	5.83%

+: genes present in this species; -: genes not present in this species

### Large parts of the *S. calchasi* transcriptome are differentially expressed in sporozoites, merozoites and bradyzoites

**Fig 3:**
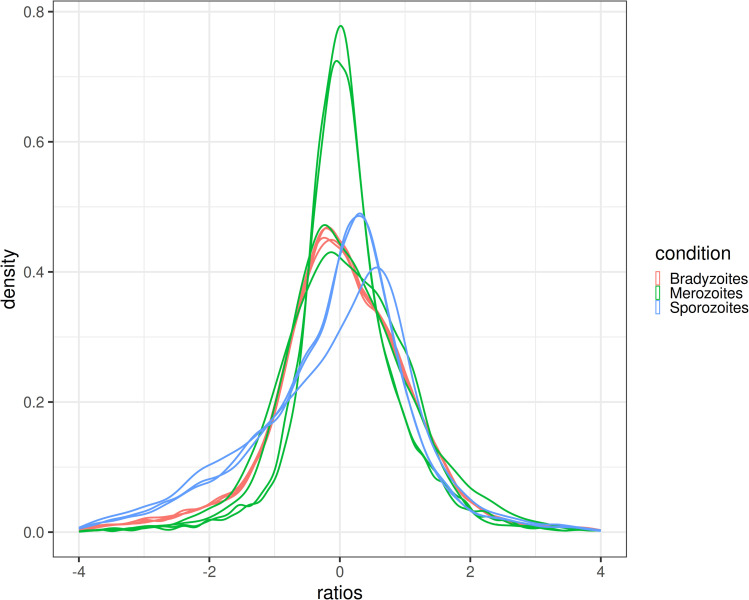
Quality assessment of RNA-seq data from *S. calchasi* bradyzoites, merozoites and sporozoites. The gene ratios between each gene and the average gene peak around zero if the majority of genes are not differentially expressed. Most *S. calchasi* samples show a bias from the expected peak at zero. This could affect the results of the differential gene expression analysis. The bandwidth was selected using the rule-of-thumb method. The number of genes in each sample ranged from 8,203 in Mero_2 to 8,884 in Mero_1.

**Fig 4:**
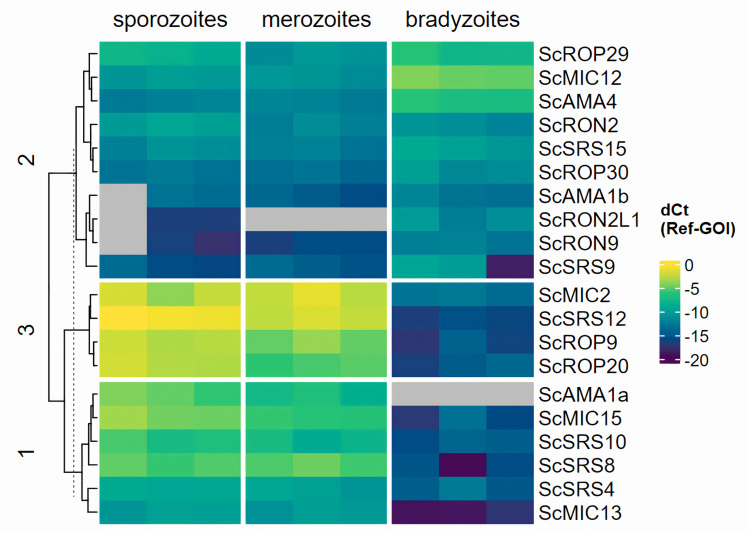
Heatmap of dCt analysis of selected SPD encoding genes in the *S. calchasi* genome. dCt values are calculated as mean (Ct[reference genes]) - Ct[gene of interest]). Gray cells mark PCRs with no measured Ct value. The clustered genes revealed three major clusters. Clusters #1 (bottom) and #3 (middle) contain genes being expressed at higher levels in sporozoites and merozoites compared to bradyzoites with a more pronounced difference in cluster #3. Cluster #2 (top) contains genes with slightly higher expression in bradyzoites.

Differential gene expression analyses with DESeq2 require the majority of genes not to be differentially expressed. The analysis of the *S. calchasi* RNA-seq samples showed a bias between the samples, as shown in Fig 3. It is expected to see all samples peak at zero, whereas some of the *S. calchasi* samples peaked at around -0.5 or 0.5. This might be an indicator that the DESeq2 analysis overestimated the number of differentially expressed genes. Additional methods were applied to support the findings of differential gene expression. To confirm RNA-seq data, 20 selected SPD genes that were differentially expressed according to DESeq2 analysis were tested by qPCR. For dCt analysis, 18SrRNA and GAPDH-1 genes were selected as reference genes as they showed best congruence of Ct values in *S. calchasi* sporozoite, merozoite and bradyzoite cDNA. Hierarchical clustering of these 20 genes confirmed TPM values by revealing two clusters of genes being expressed in higher levels in sporozoites and merozoites and one cluster of genes with higher expression levels in bradyzoites (Fig 4). Although the difference in expression in the last cluster was not as pronounced, most bradyzoites showed significantly higher expression levels by student’s t-test in all tested genes (S1 File). Additionally, *in situ*-hybridization of four SRS encoding genes (ScSRS4, 8, 10, and 12) in host tissues again confirmed RNA-seq and qPCR data regarding differential expression in merozoites and bradyzoites. In conclusion, although RNA-seq data needed to be interpreted carefully, TPM values could be confirmed by two further methods of measuring gene expression and therefore were considered to be reliable.

Of 9,716 genes detected in the *S. calchasi* transcriptome, 4,162 were significantly differentially expressed (p < 0.01) in bradyzoites compared to merozoites. In bradyzoites and sporozoites, 5,404 genes showed different transcript levels. Least expression differences were observed in merozoites compared to sporozoites with 3,356 differentially expressed genes. Approximately the same number (4,111 of 6,784) was differentially expressed when comparing *S. neurona* merozoites to schizonts [24] indicating a high level of stage-specific gene expression in *Sarcocystis* species. The number of significantly up- or down-regulated genes in bradyzoites, merozoites and sporozoites are summarized in Table 3.

**Table 3:** Differentially expressed genes in *S. calchasi* bradyzoites, merozoites and sporozoites. DESeq2 analysis on Salmon-estimated counts.

No. of genes	Compared stages	p-value	Log2FC	Up-regulated stage
1,207	B – M	< 0.001	2	B
2,204	B – M	< 0.01	1	B
838	B – M	< 0.001	2	M
1,958	B – M	< 0.01	1	M
1,616	B – S	< 0.001	2	B
2,671	B – S	< 0.01	1	B
2,004	B – S	< 0.001	2	S
2,733	B – S	< 0.01	1	S
1,313	M – S	< 0.001	2	S
2,022	M – S	< 0.01	1	S
409	M – S	< 0.001	2	M
1,334	M – S	< 0.01	1	M

B: bradyzoites; M: merozoites; S: sporozoites; Log2FC: log2 fold change

### SRS encoding genes are expressed either in sporozoites and merozoites or in bradyzoites

**Fig 5:**
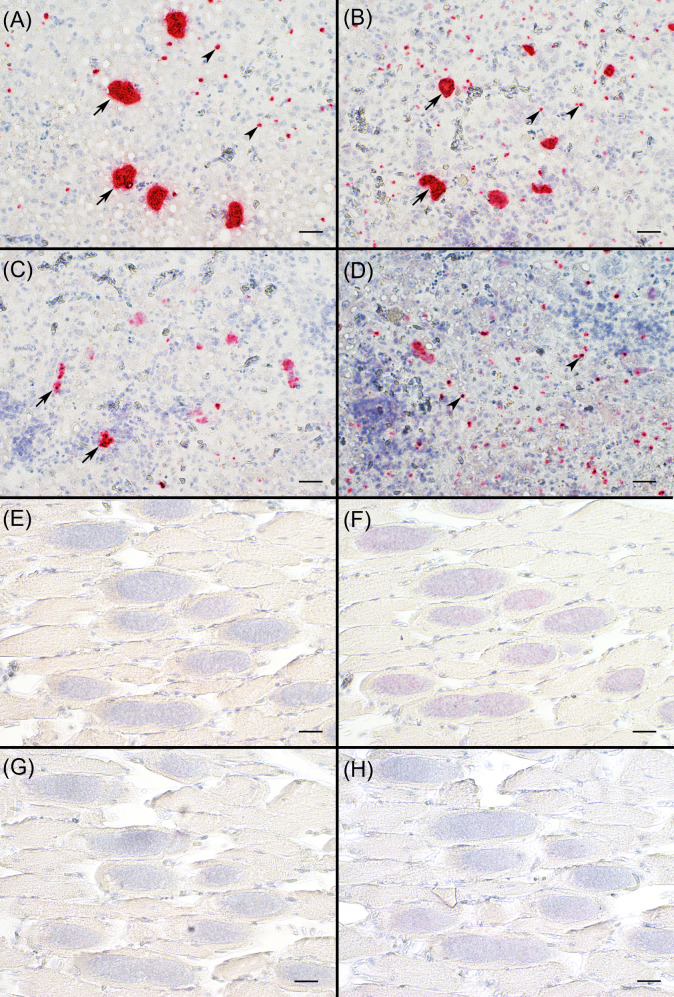
Microscopical tissue localization of ScSAG1-4 mRNA via *in situ-*hybridization. A - D: *Sarcocystis calchasi* merozoites and schizonts in the liver at dpi 10. ScSAG1 (A) and ScSAG2 (B) were strongly expressed in schizonts (arrows) as well as in merozoites (arrowheads). Weaker signals were detected for ScSAG3 mainly in schizonts (C). In contrast, ScSAG4 was predominantly expressed in merozoites (D, arrowheads). E – H: While *Sarcocystis* mRNA encoding ScSAG2 was weakly detected in few bradyzoites within muscle cysts at dpi 59 (F), mRNA encoding ScSAG1, -3 or -4 was not detected in parasitic cysts at all (E, G, H). All: *in situ-*hybridization with Fast Red as chromogen (red) and hematoxylin as counterstain (blue). Scale bars, 40 µm.

**Fig 6:**
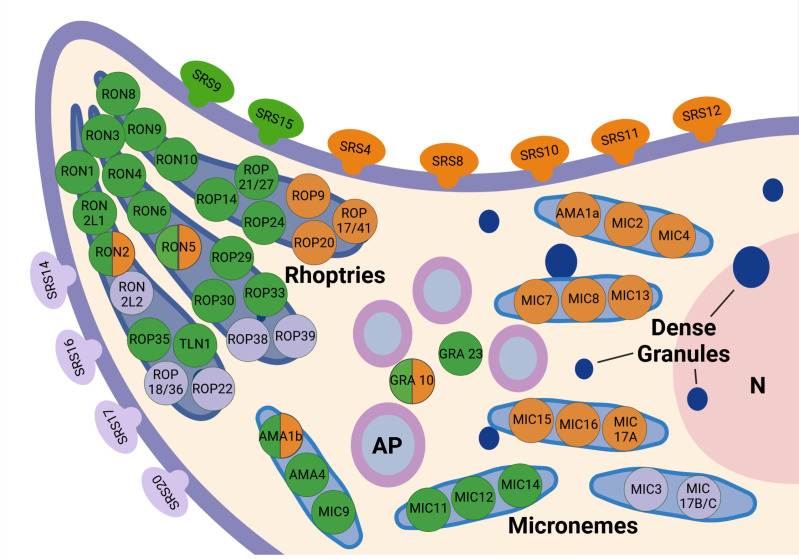
Expression patterns of *S. calchasi* SPDs. The majority of SRS and MIC encoding genes were expressed predominantly in sporozoites and merozoites while genes of rhoptry proteins were mainly expressed in bradyzoites. The expression level of RON encoding genes was generally low although detected mostly in bradyzoites. Green: genes mainly expressed in bradyzoites; orange: genes mainly expressed in sporozoites and merozoites; purple: genes not or very low expressed in bradyzoites, sporozoites and merozoites; N = Nucleus; AP = Amylopectin; Created in BioRender. Maier-Sam, K. (2025) https://BioRender.com/e35w735.

In the *S. calchasi* genome 11 SRS encoding genes were identified. The 11 SRS sequences included homologs of genes encoding immunodominant proteins in *S. neurona*, SnSAG1 (SnSRS10), SnSAG2 (SnSRS8), SnSAG3 (SnSRS12), and SnSAG4 (SnSRS4). Transcripts of the corresponding homologs in *S. calchasi* (ScSAG1, ScSAG2, ScSAG3, and ScSAG4) were abundant in merozoites and sporozoites but not or very low expressed in bradyzoites. Expression levels were determined by TPM values from RNA-seq and dCT values from qPCR analysis (S2 Table; Fig 4). Highest expression levels were detected for ScSAG3, followed by ScSAG2 and lowest expression was measured for ScSAG4 and ScSAG1. RNA-seq and qPCR data agreed with each other except for the expression of ScSAG1 in sporozoites detected by qPCR but not by RNA-seq. This order of expression intensity was contrasted by *in situ*-hybridization data from liver tissue with schizonts and free merozoites (Fig 5). The strongest signal was detected in the slides hybridized with the ScSAG1 and ScSAG2 probes. ScSAG1 and ScSAG2 mRNA was localized to schizonts as well as free merozoites. Weaker signals were obtained with the ScSAG3 probe mainly in schizonts while the ScSAG4 probe yielded weak signals from free merozoites. In bradyzoites, ScSAG1, ScSAG2, ScSAG3, and ScSAG4 expression was not or at a very low level detected by RNA-seq, qPCR and *in situ*-hybridization. Of the remaining *S. calchasi* SRS encoding genes, ScSRS11 was also found expressed by sporozoites and merozoites while ScSRS9 and ScSRS15 were predominantly expressed in bradyzoites. Four further SRS sequences (ScSRS14, ScSAG16, ScSAG17, ScSAG20) were not expressed in all three invasive stages (Fig 6).

### Genes associated with host cell invasion are well conserved in *S. calchasi*

In addition to SRS encoding genes, sequences of numerous factors of the host cell invasion machinery of apicomplexan parasites were identified in the *S. calchasi* genome. Two paralogs of AMA1 (ScAMA1a and ScAMA1b) as well as homologs to RON2, RON4, RON5, and RON8 were identified in *S. calchasi* suggesting that the invasion process is conserved. Of the sporozoite and bradyzoite associated *T. gondii* paralogs, AMA4, RON2_L1_, and RON2_L2_ homologs were identified in the *S. calchasi* genome. Stage-specific expression patterns of *S. calchasi* genes associated with host invasion differed from *Toxoplasma* and *S. neurona*. ScAMA1a and ScAMA1b whose *S. neurona* orthologues had not been found expressed in S*. neurona* merozoites (23) were differently expressed in *S. calchasi* invasive stages (Fig 4). ScAMA1a showed high expression levels in sporozoites and merozoites but no expression in bradyzoites. Expression of ScAMA1b could not be evaluated conclusively as RNA-seq data differed slightly from qPCR data. While RNA-seq implied exclusive expression of ScAMA1b in bradyzoites, qPCR detected ScAMA1b transcripts in sporozoites and merozoites without significant difference to the higher expression level in bradyzoites. ScAMA4 was expressed predominantly in bradyzoites. *Sarcocystis calchasi* RON sequences showed very low expression levels in bradyzoites with some expression of ScRON5 and ScRON2 also in sporozoites and merozoites (Figs 4 and 6).

*Sarcocystis calchasi* homologs of all RON encoding genes described in *T. gondii* were identified including homologs to RON1 and RON10 which had not been found in *S. neurona* [23].

In *S. calchasi*, homologs to CRMPA and CRMPB as well as to MIC15 and TSP-1 were annotated in its genome. While CRMPB and MIC15 were mainly expressed in *S. calchasi* sporozoites and merozoites, CRMPA and TSP-1 were also expressed in bradyzoites. Two other factors associated with rhoptry secretion and therefore essential for host cell invasion in *T.gondii* are micronemal proteins MIC8 [66] and the claudin-like apicomplexan microneme protein (CLAMP) [67]. Homologs of genes encoding for both proteins in *S. calchasi* had very low expression levels in all three invasive stages.

### MIC encoding genes show a similar expression pattern to SRS genes

From 20 MIC sequences described in *T. gondii*, 14 MIC sequences were identified in the genome of *S. calchasi*. The set of MIC encoding genes resembles that in *S. neurona* except for M2AP and MIC10 (absent in *S. calchasi*) and MIC11, MIC17A and MIC17B/C (absent in *S. neurona*). Regarding expression of MIC encoding genes in *S. calchasi* invasive stages, a clear distinction was evident between MIC genes being expressed predominantly in sporozoites and merozoites (ScMIC2, ScMIC4, ScMIC7, ScMIC8, ScMIC13, ScMIC15, ScMIC16, ScMIC17A) and those MIC genes that were expressed mainly in bradyzoites (ScMIC9, ScMIC11, ScMIC12, ScMIC14) (Fig 6). Expression levels obtained from selected MIC sequences (ScMIC2, ScMIC12, ScMiC13, ScMIC15) as detected by qPCR (Fig 4) confirmed RNA-seq data. Two further MIC genes (ScMIC3 and ScMIC17B/C) which showed low sequence homology to their *T. gondii* counterpart were not expressed in any of the three stages, possibly suggesting a potential role of these MICs in intracellular development.

### Few ROP and GRA encoding genes were annotated in the *S. calchasi* genome

In *S. calchasi*, 14 ROP homologs were identified. Nine of these homologs (ScROP17/41, ScROP20, ScROP21/27, ScROP29, ScROP30, ScROP33, ScROP35, ScROP38, ScROP39) are classified in *T. gondii* as known or likely catalytic kinases, one (ScROP22) is classified as pseudokinase and one (ScROP24) as kinase with potential nonchanonical mechanism [68]. ScROP18/36 shares equal homology to ROP18 (a kinase in *T. gondii*) and ROP36 (a pseudokinase in *T. gondii*). The function of the *T. gondii* homolog of the remaining two ROPs (ScROP9, ScROP14) remains unknown [68]. In *S. neurona*, 18 homologs of ROP encoding genes have been described [23,24], five of which being absent from *S. calchasi* (ROP19, ROP26, ROP28, ROP37, ROP40). The sequence of another soluble rhoptry protein in *T. gondii*, the metalloprotease Toxolysin-1 (TLN1), was annotated in the *S. neurona* and the *S. calchasi* genome. Overall, the total number of ROP genes in *Sarcocystis* spp. appeared rather low compared to *T. gondii* or *N. caninum* [68]. Transcripts of most ROP genes (n=8) were found predominantly in bradyzoites although two (ScROP35, ScTLN1) had very low transcript levels. Three ROP genes (ScRO9, ScROP17/41, ScROP20) were mainly expressed in sporozoites and merozoites. Four ROP genes were not expressed in either of the three stages (Fig 6).

In *S. neurona*, 4 homologs to GRA encoding genes have been identified [23,24], two of which (GRA10 and DG32 syn. GRA23/17) were annotated in the *S. calchasi* genome. Expression levels of ScGRA10 were equally moderate in sporozoites, merozoites and bradyzoites while DG32/GRA23/17 was expressed almost exclusively by bradyzoites.

### Genes associated with the inner membrane complex are well conserved and do not exhibit a distinct expression pattern

In *S. calchasi* homolog genes of most alveolins were identified. In total, homolog genes of 33 IMC proteins and all four IMC sub-compartment proteins (ISP) were present in the *S. calchasi* genome. Putative homolog genes of glideosome-associated proteins GAP40, GAP45, GAP50 and GAP70 were annotated in the *S. calchasi* genome. Other IMC proteins that are not associated to either alveolins or the glideosome are PhIL1, which is located at the apical end of the IMC [69], and MORN1 which links the IMC to the cytoskeleton [35]. Homolog genes to PhIL1 and MORN1 were also identified in the *S. calchasi* genome. The occurrence of genes encoding for IMC proteins in *S. neurona* and *S. calchasi* seems to be congruent with few exceptions (e.g., absence of GAP70 in *S. neurona*). Expression levels of genes encoding IMC proteins in *S. calchasi* sporozoites, merozoites and bradyzoites varied from genes being highly expressed in all three stages (e.g., IMC20, IMC39), genes highly expressed predominantly in a single stage (e.g., ScIMC13 in merozoites or ScPhIL1 in bradyzoites) and genes with overall very low TPM values (e.g., ScIMC5, ScIMC15).

## Discussion

*Sarcocystis* species show several differences in their life cycle, their intracellular anatomy and their life cycle compared to the well understood sister taxon *Toxoplasma gondii*. These differences are reflected on the molecular level in a different setup of specific pathogenesis determinants (SPD) as has been demonstrated in *S. neurona* [23]. To better understand *Sarcocystis* species host invasion properties during their life cycle, we sequenced and characterized the *S. calchasi* genome, identified homologous sequences and performed gene expression analyses on three *S. calchasi* invasive stages. *Sarcocystis calchasi* sporozoites and bradyzoites were extracted out of their natural hosts and merozoites were propagated in primary cell culture, also originating from a natural host species. So far, continuous passage of *S. calchasi* in cell culture remained unachieved but primary pigeon embryo liver cells and fibroblasts proved suitable cells for propagation of *S. calchasi* merozoites. The advantage of cells resembling natural host cells over more distantly related permanent cell lines has been demonstrated for other *Sarcocystis* spp. as well. For example, *Sarcocystis falcatula*-like, which parasitizes different avian intermediate hosts and opossums as definitive host, showed very poor replication in Vero cells but considerably increased release of merozoites in an avian chicken cell line [70].

The *S. calchasi* genome is the second genome of a *Sarcocystis* species to be published second to the *S. neurona* genome [23]. Like *S. neurona*, its size of 113.89 Mbp is approximately twice the size of the *T. gondii* genome. A large part of *Sarcocystis* spp. genomes (30.11 Mbp in *S. calchasi* and 31 Mbp in *S. neurona*) consists of repetitive elements, supposedly accounting for the difference in genome size compared to *T. gondii*. The number of predicted genes in *S. calchasi* (9,716 CDS) is considerably higher than in *S. neurona* SN1 (7,093 Mbp) and *T. gondii* ME49 (8,322 Mbp) which is most likely due to a large number of pseudogenes or weaknesses in gene prediction as 40.00% of genes unique to *S. calchasi* were associated with average TPM values <1. Both *Sarcocystis* species and *T. gondii* share a large core genome with 4,836 orthogroups whereas the number of common genes in *S. calchasi* and *S. neurona* is rather low (315 orthogroups) compared to genes unique to *S. calchasi* (3,409) or *S. neurona* (1,446) indicating a low number of genes unique to the genus *Sarcocystis* and consequently suggesting a high level of adaptation to its different host species. Regarding gene location, *S. calchasi* and *S. neurona* share a high level of synteny, reflecting their closer relationship compared to *T. gondii*.

The number of SRS encoding genes in *S. calchasi* (n=11) is considerably lower than in *T. gondii* (ME49 strain: 89) and roughly half the number of SRS encoding genes of *S. neurona* S3 strain. This limited set of SRS proteins seems to be conserved within the genus *Sarcocystis* and has been suggested to be linked to specific properties of the *Sarcocystis* life cycle. While *Sarcocystis* bradyzoites permanently enter a dormant phase within its cysts before being released in the definitive host’s gastrointestinal tract, *Toxoplasma* may reenter tachyzoite stage after encystation and therefore might benefit from an increased ability to alter host immune response. Additionally, *T. gondii* replicates in its definitive feline host both sexually and asexually within different host cell types and thus likely requires more diverse adhesive SRS proteins [23].

While SnSAG2, SnSAG3, and SnSAG4 are present in multiple *S. neurona* strains and are highly expressed in the merozoite stage, SnSAG1 is absent from genomes of some strains or present but not expressed in others [71]. Homologs of SnSAG2, SnSAG3, and SnSAG4 have been identified in *Sarcocystis falcatula* and other *Sarcocystis* spp. using opossums as definitive host showing high genetic variability [72]. With the detection of SnSAG2, SnSAG3, and SnSAG4 homologs in *S. calchasi,* it appears reasonable to assume that these genes may be conserved within the genus *Sarcocystis*.

Expression levels of ScSAG1–4 were high in merozoites and sporozoites whereas bradyzoites did not express any of these four SAG genes. The difference between abundance in sporozoites and absence in bradyzoites was observed for the corresponding homologs in *S. neurona* [39], making it likely that these SRS proteins play a functional role in *Sarcocystis* sporozoites and merozoites but not in bradyzoites.

Data obtained from RNA-seq, qPCR and *in situ*-hybridization agreed on the expression of ScSAG1–4 although the order of expression levels in merozoites was different between expression analyses (RNA-seq and qPCR) and *in situ*-hybridization. This difference may be due to the source of the test samples. RNA-seq and qPCR were performed on RNA extracted from free merozoites in cell culture supernatant representing a homogenous sample material with a distinct developmental stage. *In situ*-hybridization was performed on host tissues exhibiting all different stages of schizogony as well as free merozoites. ScSAGs may be expressed differently during intracellular development and in free merozoites as it was observed in *S. neurona* [24] and, therefore, detection of ScSAG mRNA in host tissue may differ notably from expression detected in free merozoites obtained from tissue culture supernatant.

Aside from ScSAG1–4, one further SRS homolog, ScSRS11, was expressed in sporozoites and merozoites while ScSRS9 and ScSRS15 are predominantly expressed in bradyzoites. The remaining SRS genes were not expressed in any invasive stage, raising the question of them playing a role in intracellular survival of the parasite.

Stage-specific composition of SRS gene expression had also been established for *T. gondii* and *N. caninum* [73,74], although Toxoplasma shows differences between tachyzoites and sporozoites [75] while *S. calchasi* seems to express the same SRS genes in sporozoites and merozoites. As sporozoites and merozoites invade host cells from the intermediate host whereas bradyzoites enter intestinal epithelial cells of the definitive host this may indicate that *Sarcocystis* spp. use different sets of SRS proteins for interaction with intermediate hosts and definitive hosts.

The invasion machinery of apicomplexans appears widely conserved and homologs of genes encoding micronemal AMA proteins and RON proteins forming the moving junction in *T. gondii* were also annotated in the *S. calchasi* genome. The difference between highly stage-specific expression levels of AMA sequences and very low expression levels of RON sequences may be due to the different distributional patterns as was observed in *T. gondii*. Specifically, AMA1 localizes on the entire surface of extracellular parasites while RON2/4/5/8 seem to be restricted to the complex forming the moving junction during invasion [27]. As RNA-seq and qPCR data of *S. calchasi* were generated from extracellular stages, ScRON2/4/5/8 expression may be higher at the time of host cell invasion.

In *T. gondii*, RON9 and RON10 form a complex which does not have an impact on *T. gondii* invasion or virulence but was suggested to be related to its development in intestinal epithelium because of its conservation in the genus *Cryptosporidium* [76]. Due to the presence of genes encoding for both rhoptry neck proteins in *S. calchasi*, a similar role can be speculated but further evidence should be provided from epithelial tissue cultures.

Micronemal proteins are supposed to mediate different functions in host cell attachment. In *T. gondii*, a complex is initially formed between MIC1, MIC4, and MIC6. In *Sarcocystis* spp. MIC4 seems to play a different role as MIC1 and MIC6 homologs are absent from both *S. neurona* and *S. calchasi*. MIC2, which is complexed in *T. gondii* with the MIC2-associated protein (M2AP), links host cell receptors with the parasitic acto-myosin motor system [27,32]. As M2AP is absent from *S. calchasi*, the role of MIC2 in *S. calchasi* remains questionable.

Expression of MIC encoding genes revealed two clearly distinguishable subsets of genes with one set of MIC genes being expressed in sporozoites and merozoites and the other being predominantly expressed in bradyzoites. This expression pattern strongly resembles that of SRS genes. As both gene families are associated with host cell attachment it may be hypothesized that host cell recognition is mediated by different subsets of factors in sporozoites and merozoites which invade intermediate host cells and bradyzoites which invade definitive host cells. The theory is supported by the observation that the number of SRS and MIC genes expressed in sporozoites und merozoites (SRS: 5, MIC: 8) is much higher than that of SRS and MIC genes expressed in bradyzoites (SRS: 2, MIC: 4). This corresponds to the greater variety of potential host species for sporozoites and merozoites (broad intermediate host spectrum in several avian orders) and the more diverse number of tissues invaded by merozoites (i.e., endothelium, hepatocytes, skeletal muscle). In contrast, bradyzoites invade intestinal epithelial cells of definitive hosts which seem so far to be limited to members of the order Accipitriformes. Thus, sporozoites and merozoites need to adapt to a wider range of host cell receptors. This may be reflected by a higher number of expressed SRS and MIC genes.

In the genomes of *S. calchasi* and *S. neurona* the number of ROP encoding genes appears to be rather low compared to *T. gondii* and *N. caninum* [23,68]. While *T. gondii* and *N. caninum* bradyzoites can be reactivated in tissue cysts and transformed into tachyzoites, *Sarcocystis* spp. remain in a dormant stage once encysted. As this reactivation likely requires more intense interaction with host cell immune response, a more diverse set of ROP proteins may be the adaptation to the more complex life cycle of *T. gondii* and *N. caninum*. Another explanation may be the lack of rhoptries and parasitophorous vacuoles in *Sarcocystis* merozoites. This is reflected by the expression pattern observed in *S. calchasi*. Most ROP genes are expressed predominantly in bradyzoites which possess rhoptries and are located within a PV in the host cell. Nevertheless, three ROP encoding genes were mainly expressed in sporozoites and merozoites indicating different functions of these proteins. Four ROP genes were not expressed in either of the three stages and two ROP genes expressed in bradyzoites (ScROP35, ScTLN1) had very low expression levels. These proteins may play a role in intracellular development as was shown in *S. neurona* where SnTLN1 is highly expressed in the schizont stage [24].

In *S. neurona* strains SN1 and SN3, homologs to 4 GRA genes are present [23,24]. Two of these were also annotated in the *S. calchasi* genome (ScGRA10 and ScDG32). In *T. gondii*, GRA23 (syn. DG32a) and GRA17 (syn. DG32b) act as pore-forming molecules in the PV membrane allowing the passage of small molecules through the PV membrane [77]. As *Sarcocystis* bradyzoites are located within a PV, a similar function is possible. Due to the very low number of annotated GRA genes and the large number of dense granules in *S. neurona* and *S. calchasi*, it appears likely that the dense granule content of *Sarcocystis* species is distinct from that of other Coccidia.

Genes encoding for proteins of the inner membrane complex (IMC) are in large parts conserved and their homologs were readily annotated to the *S. calchasi* genome. Of note, IMC-related genes are not expressed in a stage-specific manner. Their heterogenous expression patterns reflect the distinct functional roles of IMC proteins and contrast the expression of SPDs (SRS proteins and proteins of secretory organelles) which are in large parts limited to certain stages.

## Conclusion

Several components of the host attachment and invasion machinery are broadly conserved in apicomplexan parasites, and homologous genes were identified in the *Sarcocystis calchasi* genome. Differential expression patterns of specific pathogenesis determinants (SPD) associated with host cell attachment revealed subsets that were expressed in specific stages (sporozoites and merozoites) that invade intermediate hosts and others in bradyzoites that enter definitive host intestinal epithelium. Future work should focus on the identification of SPDs that are essential for host cell invasion or intracellular survival in *Sarcocystis* species and therefore could serve as potential drug or vaccine targets.

## Supporting information

S1 Table Sequences of primers targeting cDNA of selected SPD encoding genes. (XLSX)

S2 Table TPM values of selected SPD and IMC genes in *S. calchasi* bradyzoites, merozoites and sporozoites. (XLSX)

S1 File ddCt analysis of selected SPD encoding genes in the *Sarcocystis calchasi* genome. (XLSX)
